# Comparison of PCR-based high-resolution melting curve analysis and immunohistochemistry in detecting microsatellite instability in colorectal cancer: a retrospective consistency study

**DOI:** 10.7717/peerj.21607

**Published:** 2026-08-17

**Authors:** Ying Chen, Rui Lan, Qing Lv, Guiping Han

**Affiliations:** Department of Pathology, Second Affiliated Hospital of Harbin Medical University, Harbin, Heilongjiang, China

**Keywords:** Microsatellite instability, Immunohistochemistry, Colorectal cancer, PCR-HRM, Mismatch repair

## Abstract

**Objective:**

Microsatellite instability (MS I) is an important biomarker for the prognosis and immunotherapy indications of colorectal cancer (CRC). Traditional detection methods such as immunohistochemistry (I HC) and polymerase chain reaction (PCR)-based capillary electrophoresis (PCR-CE) are widely used, but there are limitations such as strong subjectivity and time-consuming result judgment. This study aims to evaluate the polymerase chain reaction-high-resolution melting curve (PCR-HRM) as a fast, high-throughput MS I detection method.

**Methods:**

Tumor samples of 251 CRC patients were analyzed. PCR-HRM and IHC methods are used to evaluate the status of MSI. If the results of PCR-HRM and IHC are inconsistent, the gold standard PCR-CE method is used for confirmation testing.

**Results:**

In this study cohort, 20.3% (51/251) of patients had MSI-H or mismatch repair defect (dMMR). PCR-HRM and IHC have excellent consistency, with an overall consistency rate of 99.20% and a Kappa coefficient of 0.971 (*P* < 0.01). Compared with IHC, the sensitivity of PCR-HRM is 98.04% and the specificity is 99.50%. In the case of inconsistency of PCR-HRM and IHC results, PCR-CE confirms the accuracy of PCR-HRM. These differences are attributed to biological phenomena, such as the presence of mutant proteins but functional inactivation (leading to false negative results in IHC) and human factors before analysis (leading to false positive results in IHC).

**Conclusion:**

PCR-HRM is a high-precision, low-cost and efficient MSI screening method. It does not require the PCR post-processing required by the traditional method, thus simplifying the workflow. In addition, it also avoids potential problems related to IHC results. Our research shows that PCR-HRM can be a powerful tool to guide the decision-making of colorectal cancer treatment.

## Introduction

Colorectal cancer (CRC) is still one of the leading causes of cancer-related incidence and mortality in the world (Patel et al., 2022). In the past decade, the characteristic analysis of microsatellite instability (MSI) and mismatch repair (MMR) defects has gone beyond its traditional use in Lynch syndrome screening (Taieb et al., 2022). With the approval of immune checkpoint inhibitors (ICIs), such as pembrolizumab and nivolumab for the treatment of unresectable or metastatic high microsatellite instability (MSI-H)/deficient mismatch repair (dMMR) solid tumors, MSI status has become a key biomarker to guide treatment decision-making (Shirav et al., 2022). This change in the diagnostic paradigm not only improves accuracy, but also achieves fast and scalable detection, thus meeting the growing demand for accurate CRC screening.

Despite the growing demand for detection and the emergence of new methods, the best method for detecting microsatellite instability is still controversial. Immunohistochemistry (IHC) is widely used as a preliminary screening method by many pathology laboratories because of its simplicity and cost-effectiveness (Wang et al., 2023; Faizee et al., 2024). However, IHC also has limitations. For example, when aminoacid substitution retains the antigenicity of protein but eliminates its catalytic activity, IHC often produces pseudo-negative results. In addition, pre-analysis factors such as tissue ischemia and fixed artifacts will also bring a certain degree of subjectivity to the interpretation of IHC results (Rekha et al., 2026; El Moutaoukkil et al., 2026). Polymerase chain reaction capillary electrophoresis (PCR-CE) is considered to be the gold standard for confirming genomic instability (MSI) (Ye et al., 2023; Shen et al., 2026). However, PCR-CE involves complex post amplification treatment and open electrophoresis, which increases the risk of pollution and is time-consuming and labor-intensive. Next generation sequencing (NGS) can provide detailed genomic profiling, but high cost and complex data analysis limit its routine use in most clinical setting, although accessibility and cost-effectiveness vary by in region and healthcare system (Mandlik, Patil & Singh, 2024; Che et al., 2026).

High-resolution melting (HRM) curve analysis has become a promising alternative method (Nikodem, Cłapa & Narozna, 2021). PCR-HRM adopts the “closed tube” method to combine the molecular accuracy of PCR with the efficiency of the automated system (Luk-In et al., 2026). By analyzing the melting behavior of the amplification product immediately after amplification, the method reduces the risk of pollution and reduces the operating cost (Jember et al., 2026). Although early studies have demonstrated the potential of PCR-HRM for MSI detection (Dietmaier & Hofstädter, 2001; Janavicius et al., 2010), large clinical cohort data comparing its performance with IHC and PCR-CE, especially for resolving discrepant cases, remain limited (Zhou et al., 2026; Ma & Gu, 2026; Moustafa, Abdalrahman & Abdelgeleel, 2026). This study aims to evaluate the diagnostic efficacy of PCR-HRM in the cohort of 251 CRC patients. This study will evaluate the consistency rate of PCR-HRM and IHC, use PCR-CE as a verification method to reverify the screening results, and evaluate whether PCR-HRM can be used as a reliable, rapid and effective routine clinical MSI testing tool.

## Materials and Methods

### Research design

This study included 251 cases of colorectal cancer diagnosed between 2022 and 2024. The research plan has been reviewed and approved by the Ethics Committee of the Second Affiliated Hospital of Harbin Medical University (Approval No.: KY2024-087). All participants signed the informed consent form before joining the group.

### Immunohistochemistry (IHC) analysis

IHC staining of tissue slices prepared by formalin fixation and paraffin embedding (FFPE) specimens, with a slice thickness of 4–5 um. After dewaxing and rehydration, use citric acid buffer with pH 6.0 for antigen repair according to the target antigen. Block endogenous peroxidase activity with 3% hydrogen peroxide, and then wash it with PBS. Sections were incubated overnight at 4 °C with primary antibodies against MLH1 (1:200; ab92312; Abcam), PMS2 (1:200; 78576; Cell Signaling Technology), MSH2 (1:200, 2017; Cell Signaling Technology) and MSH6 (1:500, 12988; Cell Signaling Technology). Then, the second anti-incubation with a horseradish peroxidase (HRP) label. Finally, use DAB (3,3′-diaminobiphenylamine) to make the color and re-dye it with hematoxylin. Dehydrate the slice with gradient ethanol series, transparent with xylene, sealed with resin-based sealing tablets, and then analyzed under a microscope.

Nuclear staining in tumor cells was considered positive. Loss of nuclear staining in tumor cells with preserved staining in internal control (normal stromal cells or lymphocytes) was defined as deficient MMR protein expression. Loss of any one of the four MMR proteins was classified as dMMR, and intact expression of all four proteins was classified as pMMR.

### PCR-CE MSI assay

According to the Maxwell CSC DNA FFPE kit, DNA was extracted from tumor and adjacent non-tumor FFPE tissue samples. The extracted DNA was then used for MSI analysis by PCR-CE. This method uses eight microsatellite markers including six mononucleotide repeat markers (BAT-52, BAT-56, NR-21, BAT-25, BAT-26 and MONO-27) and 2 pentanucleotide repeat markers to assess microsatellite stability.

### MSI analysis using high-resolution melting (HRM)

MSI in tumor tissue was evaluated using the MSI melting curve analysis kit (Wanfu Biotechnology Co., Ltd., Guangzhou, China). Genomic DNA was extracted from tumor samples. PCR amplification was performed following the manufacturer’ s instructions. The assay targets eight commonly used microsatellite loci: BAT25, BAT26, NR21, NR24, MONO-27, BAT-52, BAT-56, and CAT-25. Following amplification, HRM analysis was conducted on a real-time PCR instrument equipped with HRM capability. Samples were classified as MSI-H if at least two of the eight loci showed abnormal melting peaks indicative of mutations. Samples with one mutated locus were classified as microsatellite instability-low (MSI-L), and samples with no mutated loci were classified as microsatellite stable (MSS). HRM was performed on a real-time PCR instrument (LightCycler 480; Roche) with a melting ramp rate of 0.3 °C per second.

### Statistical analysis

In order to evaluate the performance of the PCR-HRM method in detecting MSI, we compared the results with the results with IHC as the reference standard. We have calculated key performance indicators (KPls), such as sensitivity, specificity, positive forecast value (PPV), negative forecast value (NPV) and overall consistency, as well as their 95% confidence interval (CI). We used Kappa statistics to evaluate the consistency between IHC and PCR-HRM methods. A *p* value < 0.05 was considered to be statistically significant. The following formula is used to calculate these indicators (Monaghan et al., 2021):

Sensitivity = TP/(TP + FN)

Specificity = TN/(TN + FP)

Positive predictive value = TP/(TP + FP)

Negative predictive value = TN/(TN + FN)

Overall Accuracy = (TP + TN)/(TP + TN + FP + FN)

where TP represents true positives, FN refers to false negatives, TN denotes true negatives, and FP indicates false positives.

## Results

### Patients’ characteristics

This study included 251 patients with CRC, whose demographic, clinical, and tumor characteristics are summarized in Table 1. The median age of the participants was 65 years (range 29 to 89 years), of whom 65.3% (164/251) were male. The most common tumor sites were the rectum (37.8%, 95/251) and the right colon (25.9%, 65/251). Histopathological analysis showed that adenocarcinoma was the most prevalent tumor type, accounting for 97.2% (244/251). Among these, 14.7% (37/251) were poorly differentiated, 57.8% (145/251) were moderately differentiated, and 27.5% (69/251) were well differentiated.

**Table 1:** Patient characteristics and their proportions.

**Variables**		**N**	**%**
Age		65 (29–89)	
Gender	male	164	65.3
	female	87	34.7
MMR	dMMR	51	20.3
	pMMR	200	79.7
Tumor location	rectum	95	37.8
	Right hemicolon	65	25.9
Tumor type	adenocarcinoma	244	97.2
Degree of differentiation	poorly differentiated carcinoma	143	63.84
	moderately differentiated carcinoma	56	25.00
	highly differentiated carcinoma	25	11.16
MMR status	MLH1 negative	27	71.1
	PMS2 negative	33	86.8
	MSH2 negative	13	34.2
	MSH6 negative	16	42.1
Lymphatic node transfer	yes	106	42.2
	no	145	57.8
Vascular thrombus	yes	108	43
	no	143	57
Neural invasion	yes	191	23.9
	no	143	76.1

**Notes.**

Abbreviations dMMR deficient mismatch repair pMMR proficient mismatch repair

**Figure 1:**
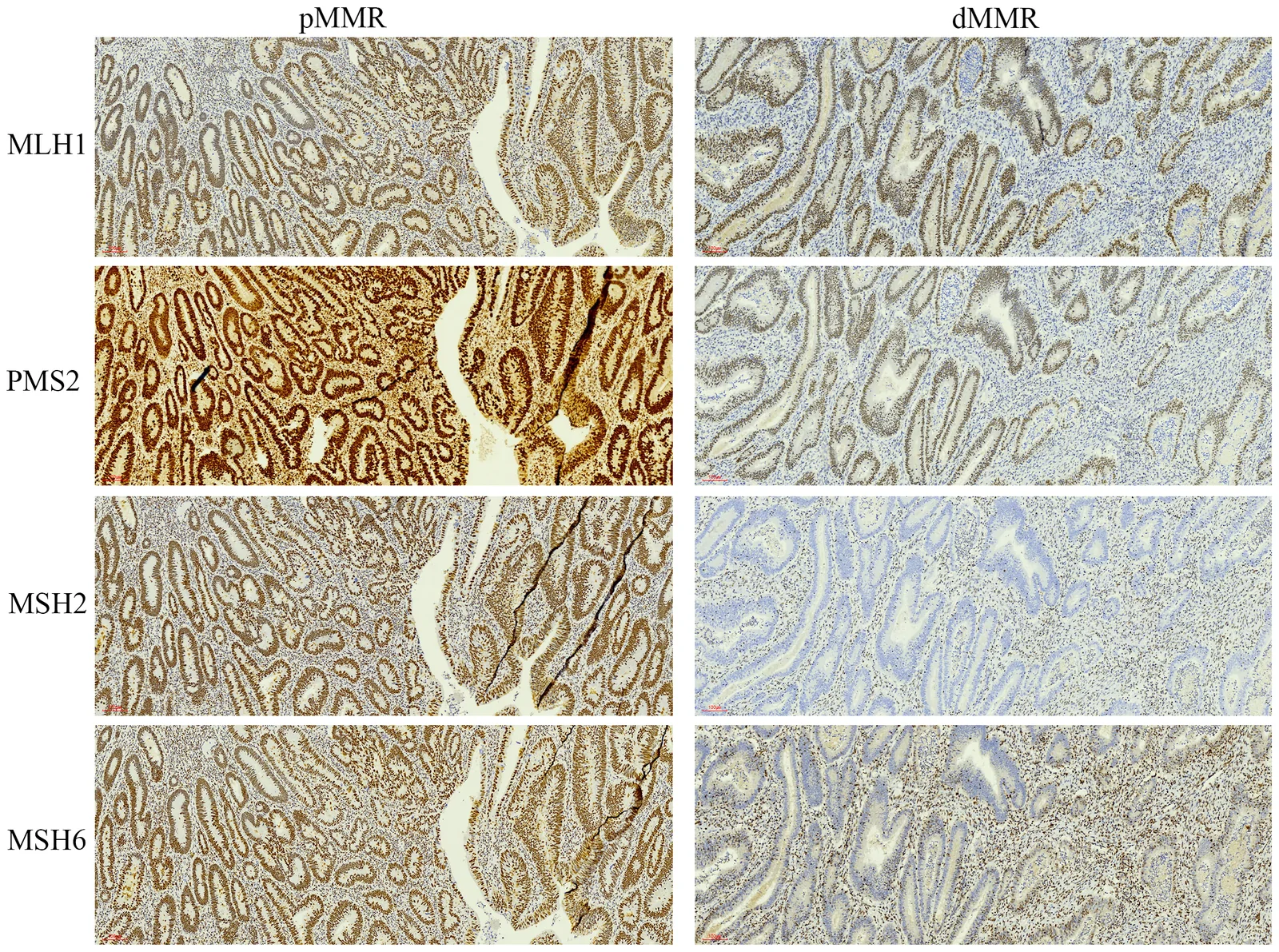
(MMR) proteins (MLH1, PMS2, MSH2 and MSH6) in pMMR and dMMR samples. On the left, the tissue with normalmismatch repair function (pMMR) is displayed, and the tissue with mismatch repair function defect (dMMR)is displayed on the right.

IHC was used to assess MMR status, and the results showed that 20.3% (51/251) of the patients had dMMR (Fig. 1). The distribution of MMR protein deficiencies among the 51 dMMR cases was as follows: MLH1 loss in 71.1% (27/51), PMS2 loss in 86.8% (33/51), MSH2 loss in 34.2% (13/51), and MSH6 loss in 42.1% (16/51). Among dMMR cases, 39.2% (20/51) showed concurrent loss of two or more MMR proteins. Most patients (93.2%, 234/251) did not receive preoperative chemoradiotherapy, while 6.8% (17/251) received preoperative chemoradiotherapy.

### Performance of PCR-HRM in MSI detection

**Figure 2:**
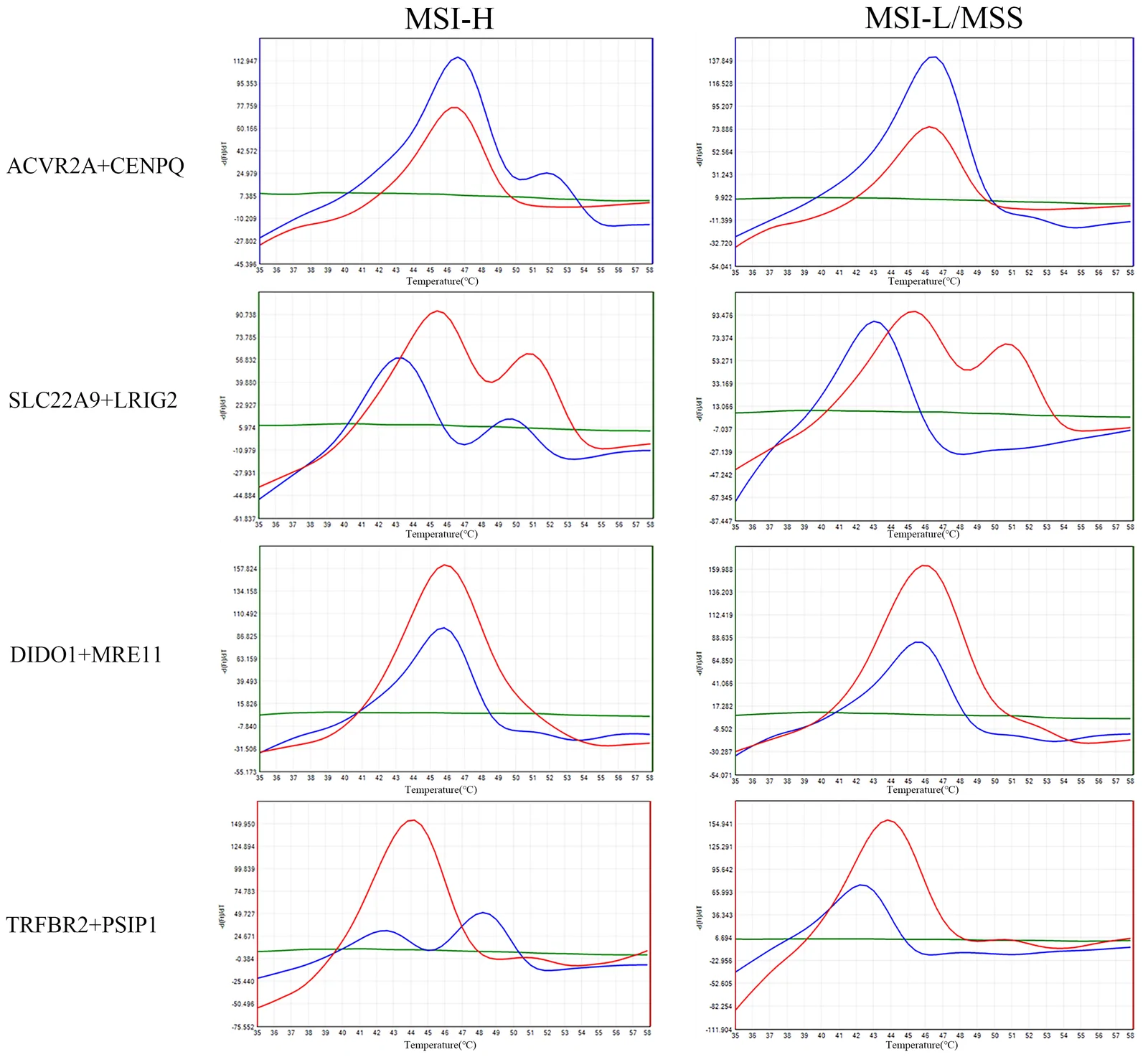
PCR-HRM melting curve analysis results: typical patterns of MSI-H and MSI-L/MSS colorectal cancer samples.

This study used PCR-HRM curve analysis and IHC methods to evaluate the MSI status of 251 CRC samples (Table 2). According to the IHC results, 51 samples (20.3%) were classified as dMMR, while 200 samples (79.7%) were identified as pMMR. The results of PCR-HRM analysis were similar, identifying 51 cases of MSI-H (20.3%) and 200 cases of MSS/MSI-L (79.7%). However, the subsequent statistical analysis showed that there were differences in the PCR-HRM and IHC results of the two groups of samples. Compared to IHC results, PCR-HRM demonstrated a sensitivity of 98.04%, a specificity of 99.50%, a PPV of 98.04%, and a NPV of 99.50% in detecting MSI-H. The overall concordance rate between the two methods was 99.20% (Table 3, Fig. 2). Table 3 presents the diagnostic performance of PCR-HRM using IHC as the initial reference standard. The two discrepant cases were resolved by PCR-CE but were not used to revise the primary IHC-based performance calculation.

Subsequently, the Kappa test was used to assess the concordance between the two detection methods. The results indicate that PCR-HRM had excellent concordance with IHC in detecting the MMR/MSI status of CRC (Kappa = 0.971, *P* < 0.01). The high Kappa value may be partly influenced by the relatively high dMMR prevalence (20.3%) in this cohort.

### Validation of inconsistent test results in two samples

**Figure 3:**
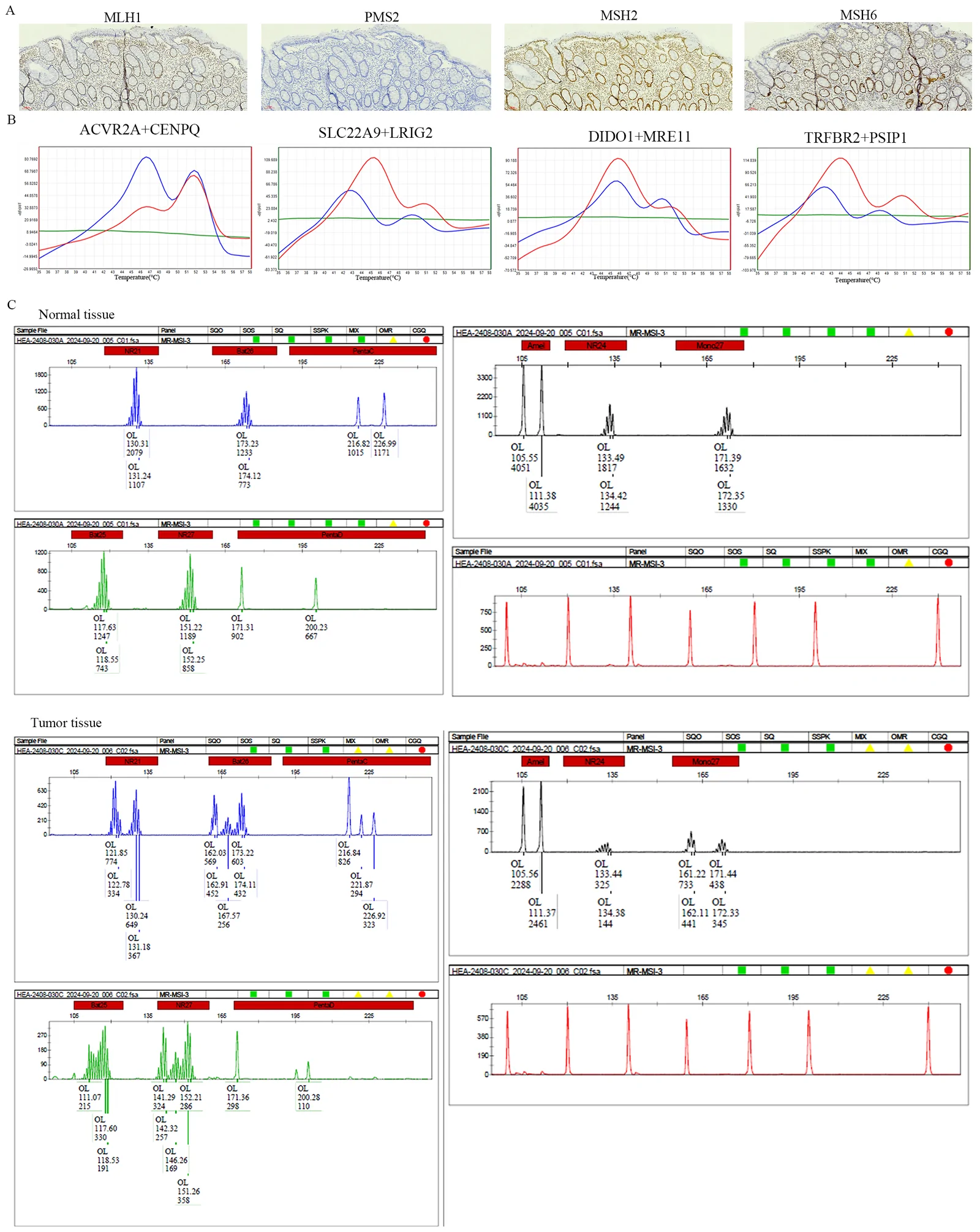
Comparison of IHC, PCR-HRM, and PCR-CE methods in detecting MSI status discrepancies in dMMR sample. To evaluate the performance of three common MSI detection assays, a dMMR tumor sample with confirmed MLH1/PMS2 loss by IHC was analyzed. (A) IHC staining of MMR proteins (MLH1, PMS2, MSH2, MSH6) was performed on formalin-fixed, paraffin-embedded (FFPE) tumor sections, with complete loss of MLH1 and PMS2 nuclear expression confirming dMMR status (×200). (B) PCR-HRM was used to assess CNV of four reference gene pairs, ensuring no copy number alterations that could interfere with MSI calling. (C) PCR-CE-based MSI testing was conducted on matched normal and tumor tissues. Normal tissue showed stable allelic peaks at all tested microsatellite loci, while the dMMR tumor exhibited extensive peak shifts and instability, consistent with MSI-H. This comparison demonstrates the high concordance between IHC and PCR-CE for MSI status determination, and the utility of PCR-HRM as a quality control assay for CNV correction.

**Figure 4:**
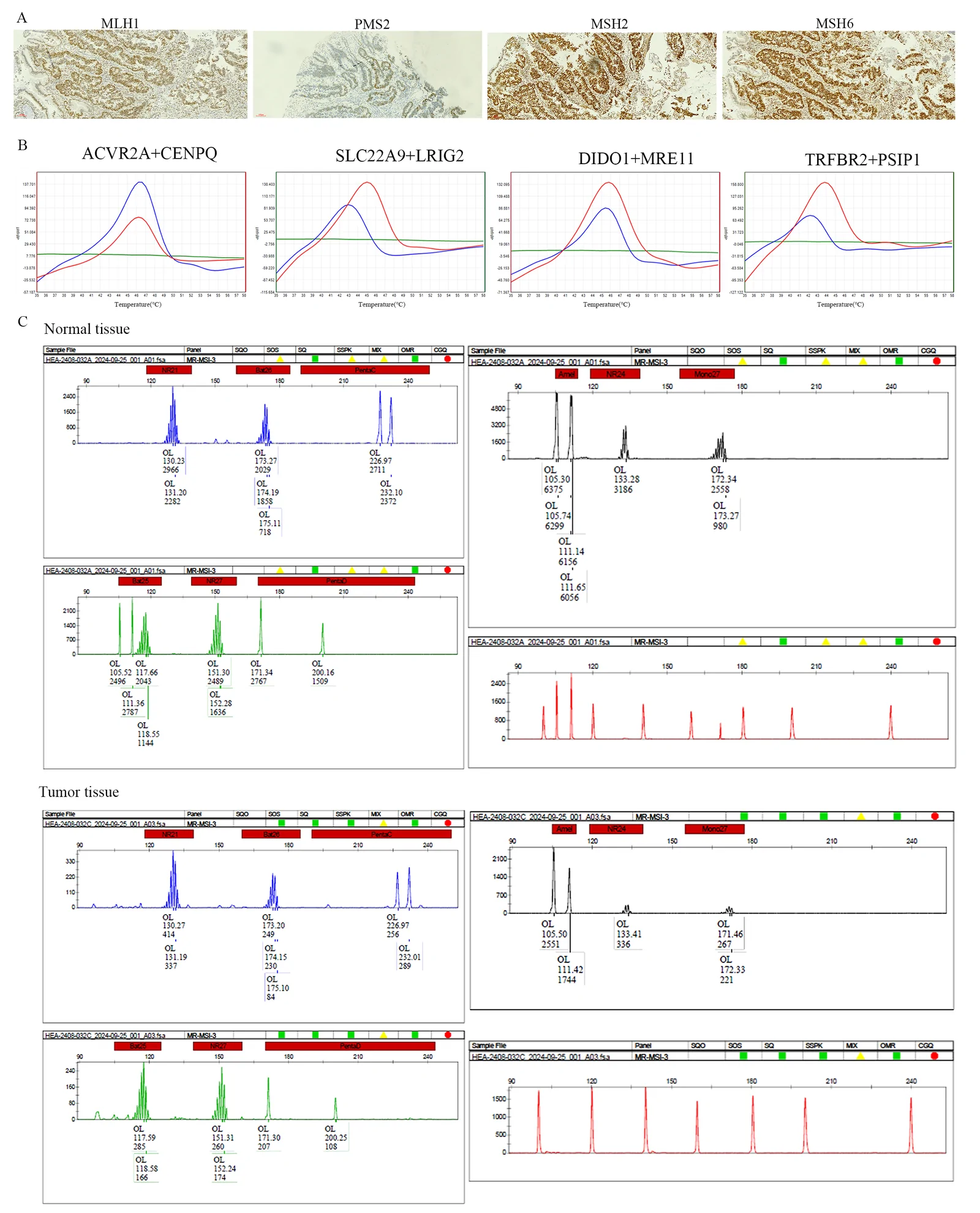
Comparison of IHC, PCR-HRM, and PCR-CE methods for detecting inconsistent MSI status in pMMR sample. To evaluate the consistency of three common MSI detection assays, a pMMR tumor sample with confirmed intact MMR protein expression by IHC was analyzed. (A) IHC staining of MMR proteins (MLH1, PMS2, MSH2, MSH6) was performed on formalin-fixed, paraffin-embedded (FFPE) tumor sections, with retained nuclear expression of all four proteins confirming pMMR status (×200). (B) PCR-HRM was used to assess CNV of four reference gene pairs, ensuring no copy number alterations that could interfere with MSI calling. (C) PCR-CE-based MSI testing was conducted on matched normal and tumor tissues. Both normal and pMMR tumor tissues showed stable, consistent allelic peaks at all tested microsatellite loci, confirming MSS status. This comparison demonstrates the high concordance between IHC and PCR-CE for MSI status determination in pMMR samples, and the utility of PCR-HRM as a quality control assay for CNV correction.

As previously noted, discrepancies were observed between the PCR-HRM and IHC methods in two of the 251 CRC patients. In the first case, the tumor was identified as pMMR by IHC but classified as MSI-H by PCR-HRM; confirmatory testing with PCR-CE yielded consistent results with PCR-HRM (MSI-H). In the second case, the tumor was identified as dMMR by IHC but classified as MSS/MSI-L by PCR-HRM, and PCR-CE confirmed the PCR-HRM result (MSS/MSI-L). To explain this discrepancy, we performed a confirmatory analysis using PCR-CE. The PCR-CE spectrum showed significant peak shifts and instability patterns, completely consistent with the PCR-HRM results (Figs. 3 and 4). The first discrepant case (IHC pMMR but molecular MSI-H) was attributed to protein retention with functional loss. The second discrepant case (IHC dMMR but molecular MSS) was attributed to pre-analytical factors such as tissue fixation artifacts. These confirmatory results suggest that the observed inconsistencies, particularly the false-negative results of IHC, may be due to uneven or weak expression of MMR proteins, or the presence of catalytically inactivated but antigenically intact mutant proteins. Therefore, these results show that PCR-HRM can be used as a fast, reliable and clinically significant CRC MSI detection method. However, its effectiveness still needs to be further verified through prospective multicenter studies to confirm its broader clinical application value and accuracy.

**Table 2:** Comparison of PCR-HRM and IHC results.

		**PCR-HRM**		**Total**
		**MSI-H**	**MSI-L/MSS**	
Immunohistochemistry (IHC)	dMMR	50	1	51
	pMMR	1	199	200
Total		51	200	251

**Table 3:** Sensitivity, specificity and consistency analyses of the PCR-HRM assay compared to the IHC assay.

	**[95% CI]**
Sensitivity	98.04% ± 3.84%
Specificity	99.50% ± 0.69%
Positive predictive value	98.04% ± 3.84%
Negative predictive value	99.50% ± 0.69%
Overall accuracy	99.20% ± 1.10%

## Discussion

The evaluation of MSI has surpassed its traditional role in Lynch syndrome (LS) screening and has become the cornerstone of precision oncology (Pussila et al., 2024; Yamamoto et al., 2024). With the approval of ICIs such as pembrolizumab to treat unresectable or metastatic MSI-H/dMMR solid tumors, accurate biomarker detection has become crucial (Shirav et al., 2022). Although the current NCCN and ESMO guidelines recommend universal screening for all CRC patients, there are still differences in the diagnostic methods chosen by different institutions. Traditional IHC is widely used because of its ease of operation and cost-effectiveness; however, its inherent limitation is that it cannot detect the lack of function in the presence of antigenically intact proteins. On the contrary, although second-generation sequencing (NGS) has high sensitivity, in many areas with limited resources, its cost is still too high to be used in conventional initial screening (Mandlik, Patil & Singh, 2024). Against this background, our research has verified that PCR-HRM is a strategic “compromise scheme”, which combines the genomic accuracy of molecular detection with the turnover time and cost structure comparable to IHC.

In the cohort of 251 CRC patients we included, we found that the prevalence of dMMR/MSI-H was 20.3%, which is slightly higher than the 15% commonly reported in Western population (Siegel et al., 2023), but consistent with recent data of the Asian population, suggesting potential regional or racial differences (Zhang & Chen, 2021; Xu et al., 2024). However, this higher prevalence should be interpreted with caution due to possible selection bias, as this was a single-center retrospective study rather than a population-based cohort. Our data emphasizes the need to establish a population-specific baseline when evaluating diagnostic effectiveness. In terms of diagnostic performance, the sensitivity of PCR-HRM detection is 98.04% and the specificity is 99.50%. The high consistency observed in this study (Kappa = 0.971) shows that the optimization of the HRM scheme effectively reduces the technical errors that previously limited the application of the technology.

The key contribution of this study lies in the molecular analysis of two inconsistent cases, thus providing important insights into the biological limitations of protein-based detection methods. One patient was identified as MSI-H by PCR-HRM and PCR-CE but showed normal nuclear staining for all four MMR proteins (pMMR) on IHC. This inconsistency is often called “function loss with protein retention”, accounting for about 5–10% of Lynch syndrome cases (Acha-Sagredo et al., 2025; Takei et al., 2024). This situation is mainly caused by missense mutations, which reduce the catalytic efficiency of the MMR enzyme complex, but will not trigger protein degradation. In this case, the antibody detects inert protein, which leads to the wrong “normal function” classification. This finding strongly supports a diagnostic algorithm in which PCR-based testing should be performed as a reflex test when MMR deficiency is clinically suspected but IHC is normal. Conversely, one case was classified as dMMR by IHC but confirmed as MSS by both molecular methods, likely due to preanalytical factors such as delayed tissue fixation or tumor heterogeneity leading to antigen loss unrelated to genetic mutation. In this case, relying solely on IHC may lead to inappropriate immunotherapy, causing the patient to suffer from immune-related adverse events without the benefits of treatment. Our results show that PCR-HRM plays a crucial arbitration role in these ambiguous situations.

We propose a clinical workflow: IHC can be used for initial screening, and PCR-HRM is recommended as a reflex test for IHC-pMMR cases with high clinical suspicion of dMMR, as well as for validation of IHC-dMMR cases. This combined strategy maximizes accuracy while maintaining efficiency.

Although PCR-CE has long been considered the gold standard for molecular biology, it requires PCR post-processing, such as gel electrophoresis or capillary separation, which increases the risk of cross-contamination and prolongs the diagnostic cycle (Nikodem, Cłapa & Narozna, 2021). Our research confirms that PCR-HRM, as a closed-tube method, does not require amplification and post-treatment. This feature not only minimizes the risk of pollution, but also significantly simplifies the laboratory workflow. Comparative studies show that compared with PCR-CE, PCR-HRM can reduce the cost of reagents by about 30–50% and save hours of technician operation time (Nikodem, Cłapa & Narozna, 2021). These logistics advantages make PCR-HRM an ideal choice for high-throughput clinical laboratories.

We admit that there are some limitations in the design of this study. Although 251 samples are sufficient to provide statistical efficacy to assess overall consistency, the number of inconsistency cases (*n* = 2) is too small to fully elucidate all potential mechanisms underlying inconsistent results between IHC and PCR-HRM. Notably, PCR-CE was only performed for discrepant cases rather than all samples, which may lead to slight overestimation of diagnostic performance. Larger multi-center cohorts are needed to capture rare MMR variants. We used a standard combination of single nucleotide markers. Although the specificity of single nucleotide markers is usually better than that of dinucleotide markers (Kim et al., 2025), the emergence of extended combinations (such as Pentaplex) deserves further comparison to determine whether the sensitivity can be slightly improved. Like all PCR-based methods, the sensitivity of HRM is affected by tumor purity. Samples with low tumor cell content (<20%) may produce MSS false negative results due to the masking effect of wild DNA in normal matrix cells. The improved detection method in the future can be combined with digital PCR (dPCR) technology to overcome this sensitivity threshold, so as to identify low-frequency MSI cloning.

This study confirmed that PCR-HRM is a robust, high-precision and clinically feasible alternative method, which can be used for MSI detection in colorectal cancer, with simpler operation, lower cost and higher clinical throughput than the traditional PCR-CE method. PCR-HRM can detect functional MMR defects omitted by IHC and is easy to operate, making it an ideal initial screening tool. Applying PCR-HRM to the routine pathological workflow will improve the accuracy of prognosis stratification and ensure that all patients who meet the conditions of immunotherapy can be correctly identified.

## Conclusion

In conclusion, this study demonstrates that PCR-HRM is a robust, highly accurate, and cost-effective method for MSI detection in colorectal cancer, effectively bridging the gap between traditional IHC and NGS/PCR-CE. By addressing the inherent limitations of IHC—specifically its inability to detect “functional loss with protein retention” resulting from missense mutations—PCR-HRM serves as a critical arbitration tool for resolving discrepant diagnostic results. Furthermore, its closed-tube format, lower reagent costs, and streamlined workflow provide significant logistical advantages over the conventional PCR-CE gold standard, making it well-suited for high-throughput clinical laboratories. We propose a combined diagnostic workflow utilizing IHC for primary screening, with PCR-HRM as a reflex test for validation in ambiguous cases. Implementing this strategy will optimize prognostic stratification and ensure the accurate identification of patients eligible for immunotherapy.

## Supplemental Information

10.7717/peerj.21607/supp-1 Supplemental Information 1 Data

10.7717/peerj.21607/supp-2 Supplemental Information 2 Codebook for categorical data

10.7717/peerj.21607/supp-3 Supplemental Information 3 MIQE Checklist

10.7717/peerj.21607/supp-4 Supplemental Information 4 STROBE Checklist

## Abbreviations

CRC: Colorectal cancer
MSI: Microsatellite instability
MSI-H: Microsatellite instability-high
MSI-L: Microsatellite instability-low
MSS: Microsatellite stable
MMR: Mismatch repair
IHC: Immunohistochemistry
HRM: High-resolution melting
PCR-CE: Polymerase chain reaction-capillary electrophoresis
FFPE: Formalin-fixed paraffin-embedded
PPV: Positive predictive value
NPV: Negative predictive value
dMMR: Deficient mismatch repair
pMMR: Proficient mismatch repair
ICIs: Immune checkpoint inhibitors
TP: True positive
FN: False negative
TN: True negative
FP: False positive

## Additional Information and Declarations

### Data Availability

The following information was supplied regarding data availability: The data is available in the Supplemental Files. The raw data is available at Figshare: Han, Guiping (2026). Comparison of PCR-Based High-Resolution Melting Curve Analysis and Immunohistochemistry in Detecting Microsatellite Instability in Colorectal Cancer: A Consistency Study. figshare. Dataset. https://doi.org/10.6084/m9.figshare.31236589.v1.
